# Effect of the Arg16Gly β_2_‐Adrenergic Receptor Polymorphism on Long‐Term Mepolizumab Response and Clinical Remission in Severe Eosinophilic Asthma: A Genotype‐Stratified, Multicenter Study

**DOI:** 10.1111/all.70071

**Published:** 2025-09-23

**Authors:** Santi Nolasco, Evelina Fagone, Raffaele Campisi, Andrea Portacci, Giulia Scioscia, Corrado Pelaia, Angelantonio Maglio, Claudio Candia, Vitaliano Nicola Quaranta, Isabella Carrieri, Alessandro Saglia, Alessandro Vatrella, Girolamo Pelaia, Carlo Vancheri, Maria Pia Foschino Barbaro, Maria D'Amato, Giovanna Elisiana Carpagnano, Nunzio Crimi, Claudia Crimi, the Southern Italy Network on Severe Asthma Therapy, Rossella Intravaia, Morena Porto, Pietro Impellizzeri, Maria Provvidenza Pistorio, Fabio Vignera, Andrea Alessia Nardo, Concetta Giannì, Silvano Dragonieri, Flogerta Sana, Valeria Pezzuto, Santina Ferulli, Massimo Triggiani, Roberta Parente, Pasquale Tondo, Piera Soccio, Donato Lacedonia, Luigi Ciampo, Antonio Franzese, Valeria Longobardi

**Affiliations:** ^1^ Department of Clinical and Experimental Medicine University of Catania Catania Italy; ^2^ Respiratory Medicine Unit Policlinico “G. Rodolico‐San Marco” University Hospital Catania Italy; ^3^ Institute of Respiratory Disease Department of Translational Biomedicine and Neuroscience University “Aldo Moro” Bari Italy; ^4^ Department of Medical and Surgical Sciences University of Foggia Foggia Italy; ^5^ Department of Health Sciences University “Magna Graecia” of Catanzaro Catanzaro Italy; ^6^ Department of Medicine, Surgery and Dentistry University of Salerno Salerno Italy; ^7^ Department of Respiratory Medicine University “Federico II” of Naples Naples Italy; ^8^ Division of Allergy and Clinical Immunology University of Salerno Fisciano Italy

**Keywords:** β_2_‐adrenergic receptor, Arg16Gly, mepolizumab, remission, severe asthma

## Abstract

**Background:**

β_2_‐adrenergic signaling promotes airway smooth muscle relaxation and limits the release of pro‐inflammatory mediators by immune cells. The rs1042713 polymorphism encodes a glycine‐to‐arginine substitution (Arg16Gly) that enhances β_2_‐receptor downregulation. We investigated the association of this polymorphism with the risk of severe eosinophilic asthma and its impact on the long‐term effectiveness of mepolizumab and clinical remission.

**Methods:**

Genotypes from 102 patients with severe eosinophilic asthma receiving mepolizumab were compared with those from 31 individuals with mild asthma and 20 healthy controls. The severe‐asthma cohort was followed for up to 24 months, and clinical data were collected at baseline and after 3, 6, 12, and 24 months of treatment. Analyses were stratified by Arg/Arg, Arg/Gly, and Gly/Gly genotypes.

**Results:**

Each additional Arg16 allele increased the odds of severe eosinophilic asthma by 2.61‐fold (95% CI 1.48–4.59; *p* = 0.0001) relative to mild asthma and by 3.61‐fold (95% CI 1.78–7.35; *p* < 0.0001) relative to healthy controls. Over 24 months of mepolizumab treatment, Arg/Arg patients had an increased risk of exacerbations (HR 2.3 [95% CI 1.03–5.20]; *p* = 0.0414) and poorer asthma control compared with Gly/Gly patients (ACT ≥ 20: 72.4% vs. 100%, *p* = 0.0308). Gly/Gly patients also experienced less decline in lung function. By month 24, each additional Gly16 allele increased the odds of achieving clinical remission by 2.86‐fold (95% CI 1.20–6.81; *p* = 0.0170), defined as no annual exacerbations, no OCS, and ACT ≥ 20, and by 3.06‐fold (95% CI 1.34–6.96; *p* = 0.0080) when including an FEV_1_ decline ≤ 5% from baseline.

**Conclusions:**

The Arg16 allele of the rs1042713 polymorphism increases the risk of severe eosinophilic asthma and may reduce the long‐term efficacy of mepolizumab, whereas the Gly16 allele appears to confer better outcomes and higher remission rates.

## Graphical abstract

This study examines the association between the rs1042713 (Arg16Gly) polymorphism of the β_2_‐adrenergic receptor and the risk of severe eosinophilic asthma, as well as its influence on mepolizumab long‐term effectiveness and clinical remission. The Arg16 allele is associated with increased risk of severe disease and potentially diminished long‐term responsiveness to mepolizumab. The Gly16 allele correlates with better outcomes and higher remission rates. ACT, Asthma Control Test; Arg, arginine; FEV1, forced expiratory volume in one second; Gly, glycine; OCS, oral corticosteroids; SNP, single nucleotide polymorphism.

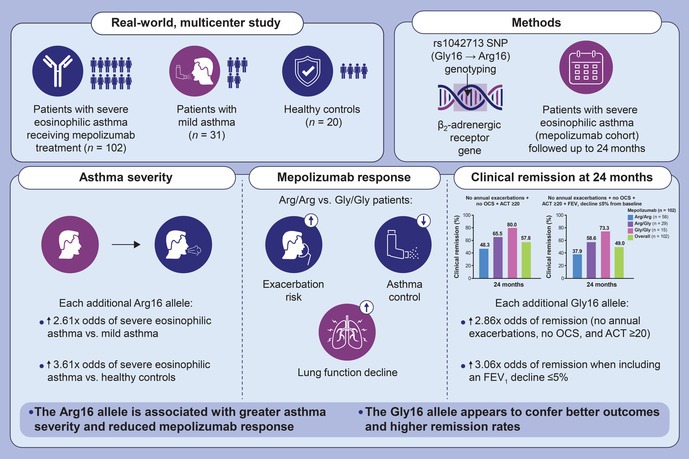

**Abbreviations**
ACT: Asthma Control Test
Arg: arginine
BMI: body mass index
FEF_25%–75%_
: forced expiratory flow between 25% and 75% of FVC
FeNO: fractional exhaled nitric oxide
FEV_1_
: forced expiratory volume in one second
FVC: forced vital capacity
Gly: glycine
ICS: inhaled corticosteroid
IgE: immunoglobulin‐E
IL‐5: interleukin‐5
LABA: long‐acting β_2_‐agonist
LAMA: long‐acting muscarinic antagonist
mAb: monoclonal antibody
MCID: minimal clinically important difference
OCS: oral corticosteroids (prednisone)
SABA: short‐acting β_2_‐agonist

## 1 Introduction

Mepolizumab, a humanized monoclonal antibody (mAb) targeting interleukin‐5 (IL‐5), has demonstrated safety and efficacy in reducing exacerbations and enhancing quality of life in patients with severe eosinophilic asthma [1, 2, 3, 4, 5, 6].

The significant clinical effectiveness of mepolizumab and other monoclonal antibodies has transformed the management of severe asthma, leading to the adoption of a new ambitious therapeutic goal known as “clinical remission” [7]. Clinical remission is defined by the absence of asthma exacerbations, no need for oral corticosteroids (OCS), adequate symptom control, and stable or normalized lung function [8, 9, 10, 11, 12]. However, only about one‐third of patients treated with mepolizumab achieve clinical remission [13, 14, 15, 16, 17, 18]. Indeed, in a recent meta‐analysis [11], only 38% of patients treated with biologics achieved clinical remission, defined by the absence of exacerbations, elimination of OCS, and controlled asthma symptoms; this figure dropped to 30% when stabilization or normalization of lung function was also included. Suboptimal responses to biologics suggest that multiple factors, including genetic variants [19], may influence treatment outcomes.

The single nucleotide polymorphism rs1042713 of the β_2_‐adrenergic receptor gene encodes a glycine‐to‐arginine substitution at amino acid position 16 (Arg16Gly) which is associated with enhanced β_2_‐receptor downregulation and tachyphylaxis [20], resulting in diminished receptor function. This variant is relatively common, with an allele frequency of approximately 40% (±15% depending on the ethnicity) worldwide [21, 22, 23, 24], and its association with asthma risk has been particularly noted in Arab and Hispanic‐Latin populations [25]. Furthermore, the Arg/Arg genotype has been linked to more severe asthma, as individuals with severe allergic asthma have been reported to be approximately four times more likely to have the Arg/Arg genotype compared to those with mild‐to‐moderate asthma [22].

Over the last two decades, this polymorphism has been extensively studied, although results have not always been consistent. The BARGE trial reported reduced morning peak expiratory flow (PEF) with regular short‐acting β_2_‐agonists (SABA) use in Arg16 homozygotes (Arg/Arg) compared to Gly16 homozygotes (Gly/Gly) [26]. Similarly, earlier analyses suggested decreased PEF among Arg/Arg patients treated with long‐acting β_2_‐agonist (LABA) [27]; however, later genotype‐stratified clinical trials did not replicate this finding [28, 29]. Nevertheless, one trial demonstrated reduced bronchoprotection against methacholine with LABA use, even when combined with inhaled corticosteroids (ICS) [28]. Additionally, a large cohort study indicated that the Arg/Arg genotype was associated with a steeper lung function decline, irrespective of LABA and/or ICS use [30]. In children and young adults, the Arg16 allele is associated with an increased exacerbations risk if exposed to daily SABA or LABA [31, 32].

Notably, the β_2_‐adrenergic receptor is expressed not only in airway smooth muscle but also in epithelial cells and various immune cells, including group 2 innate lymphoid cells (ILC2) [33] and eosinophils [34]; its activation suppresses the release of pro‐inflammatory mediators [33, 34] and limits eosinophils recruitment [34]. Thus, rs1042713 may contribute to both reduced smooth muscle susceptibility to adrenergic stimuli and enhanced eosinophilic inflammation.

In this study, we aimed to assess the association of this polymorphism with the risk of severe eosinophilic asthma compared with patients with mild asthma and healthy controls, and to evaluate its influence on the long‐term effectiveness of mepolizumab and the likelihood of achieving clinical remission.

## 2 Methods

### 2.1 Study Design

We conducted a longitudinal, multicentre, case–control study across seven tertiary care facilities within the “Southern Italy Network on Severe Asthma Therapy” between November 2017 and November 2022 (see Supporting Information for a complete list of participating centers). Patients with severe eosinophilic asthma constituted the “case”, while patients with mild asthma and healthy individuals served as “controls”. Within the severe eosinophilic asthma cohort, we further explored potential genetic influences on treatment response and remission status by comparing subgroups according to Arg16Gly genotype (Arg/Arg, Arg/Gly, Gly/Gly).

This study was conducted in accordance with Good Clinical Practice standards and adhered to the Declaration of Helsinki. The research protocol was approved by the “Catania 1” Ethics Committee of the Policlinico University Hospital (Approval Numbers 14/2020/PO—March 24, 2020 and 33/2020/PO—April 6, 2020, Catania, Italy) as well as the local ethics committee of each study site. All participants provided written informed consent.

### 2.2 Study Populations

The severe eosinophilic asthma cohort included adults (≥ 18 years old) with confirmed adherence to maintenance therapy and a diagnosis of severe asthma according to the European Respiratory Society/American Thoracic Society (ERS/ATS) guidelines [35]. At baseline, participants had a blood eosinophil count ≥ 150 cells/μL and a history of at least ≥ 300 eosinophils/μL and ≥ 2 exacerbations in the previous year despite high doses of ICS plus LABA (GINA step 5) [36]. These patients received subcutaneous mepolizumab (100 mg, once every 4 weeks) for at least 24 months. Patients with inducible laryngeal obstruction (ILO) or vocal cord dysfunction (VCD) were excluded from the study due to the risk of misdiagnosis and potential confounding of the accurate assessment of true asthma control and treatment response.

Patients with mild asthma, defined as well‐controlled asthma on GINA step 1–2 therapies [36], and healthy controls were enrolled from the Respiratory Medicine Unit of the Policlinico “G. Rodolico—San Marco” University Hospital in Catania.

### 2.3 β_2_‐Adrenergic Receptor Genotyping

Five milliliters of venous blood were collected from each participant for genotype analysis. Samples from clinical sites were stored at −20°C, transported on dry ice in temperature‐controlled containers, and inspected for integrity upon arrival before use. All DNA extraction and genotyping experiments were performed at the University of Catania, using a standardized protocol based on the method described by Martinez et al. [37]. Details about the genotyping protocol are provided in the Supporting Information.

### 2.4 Data Collection

A shared database was created for data collection, developed in collaboration with and approved by all participating centers. Demographic and clinical characteristics were collected before mepolizumab initiation (baseline) and at 3, 6, 12, and 24 months. Severe asthma exacerbations were defined as disease worsening requiring three or more days of treatment with systemic corticosteroids (or a doubling of the prednisolone equivalent dose if already on OCS) [38]. Exacerbations treated with cycles of corticosteroids less than 7 days apart were considered as the same episode.

Asthma control was assessed using the Asthma Control Test (ACT) [39, 40]. The total score of the test is 25, with a score ≥ 20 indicating well‐controlled asthma. The proportion of patients achieving an increase ≥ 3 (minimal clinically important difference, MCID) was also assessed [41].

Pulmonary function tests were performed according to the ERS/ATS guidelines [42]. Data on pre‐bronchodilator forced expiratory volume in one second (FEV_1_% and L), forced vital capacity (FVC%), FEV_1_/FVC%, and forced expiratory flow between 25% and 75% of FVC (FEF_25%–75%_) were collected. Changes in OCS doses (prednisone equivalent dose) were registered. Patients requiring as‐needed SABA were assessed. Eosinophil levels in peripheral blood were measured at each time point. The fractional exhaled nitric oxide (FeNO) was analyzed according to the ATS/ERS recommendations [43].

### 2.5 Definitions of Clinical Remission and Sustained Remission

Clinical remission was evaluated and reported in accordance with expert consensus [8, 9, 10, 11, 12]. The *main definition* (3‐component) encompasses the key domains of absence of severe asthma exacerbations, no OCS use for asthma treatment, and controlled symptoms. A *secondary definition* (4‐component) also included stabilization of lung function [44], as follows:

1. *Main definition*: No annual severe exacerbations [38] + no maintenance OCS use in the past 6 months + ACT ≥ 20
2. *Secondary definition*: No annual severe exacerbations [38] + no maintenance OCS use in the past 6 months + ACT ≥ 20 + FEV_1_ decline ≤ 5% from baseline;

Furthermore, we conducted sensitivity analyses applying an FEV_1_ ≥ 80% predicted and an FEV_1_ improvement of +100 mL from baseline. We also explored a decline in FEV_1_ < 100 mL from the best value within the first 12 months [44]. Sustained remission was defined as achieving clinical remission after 12 months and maintaining this status continuously up to month 24.

### 2.6 Statistical Analysis

Based on prior data [21, 22, 23, 24], we calculated that a sample size of 100 severe eosinophilic asthma patients, 30 mild asthma patients, and 20 healthy controls would achieve 85% power (*α* = 0.05) to detect differences in Arg/Arg genotype prevalence.

Data were tested for normality using Q‐Q plots. Continuous variables are reported as mean and standard deviation (SD) if normally distributed, or as median and interquartile range (IQR) if non‐normally distributed. Categorical variables are presented as numbers (*n*) and percentages (%).

The Hardy–Weinberg equilibrium was assessed in mild asthmatics and healthy controls [45] by exact test. Multinomial logistic regression was performed to assess the association between Arg16Gly genotype (coded additively as Gly/Gly = 0, Arg/Gly = 1, Arg/Arg = 2 to reflect the apriori hypothesis that the Arg allele increases disease severity) [46] and disease status (severe eosinophilic asthma vs. mild asthma and healthy controls). Models were adjusted for age, sex, and body mass index (BMI). Odds ratios (ORs) and 95% confidence intervals (95% CIs) were calculated to estimate the effect of each additional Arg16 allele on disease risk.

For baseline between‐group comparisons, ANOVA or the Kruskal–Wallis test was used, as appropriate. Within‐group comparisons of continuous outcomes from 3 to 24 months versus baseline were performed using Dunnett's test or the Friedman test followed by Dunn's post hoc tests. Cox proportional hazards regression models were fitted to generate Kaplan–Meier curves for the time to asthma exacerbations. The Mantel–Haenszel test was used to estimate hazard ratios (HRs) and 95% CIs. A linear mixed‐effects model was used to compare changes in parametric variables between groups, including patients as a random effect and genotype (coded additively as Gly/Gly = 0, Arg/Gly = 1, Arg/Arg = 2), age, and time (12 and 24 months) as fixed effects. Bonferroni post hoc correction was applied for multiple comparisons. The Jonckheere–Terpstra test, also with Bonferroni post hoc, was used to compare changes in nonparametric variables at 12 and 24 months. Mean or median differences and 95% CIs were calculated to assess treatment effects. The Cochran‐Armitage test for trend or the Fisher exact test, with or without Freeman–Halton extension, was used for comparisons of categorical variables when appropriate.

To identify factors associated with clinical and sustained remission, we fitted separate binary logistic‐regression models, each using remission status (yes/no)—defined according to the *main* and *secondary definitions*—as the dependent variable and genotype as the key explanatory variable. Given our hypothesis that the Gly16 allele may confer a protective effect, we reversed the additive genotype coding specifically for this analysis (Arg/Arg = 0, Arg/Gly = 1, Gly/Gly = 2) [46]. All models were adjusted for study center, age, sex, BMI, smoking history, baseline annual exacerbation rate, current inhaled therapy, daily oral corticosteroid use, leukotriene receptor antagonist therapy, and baseline blood eosinophil count. Results are presented as adjusted ORs with 95% CIs.

Statistical analysis was performed using Prism version 10.1.0 (GraphPad Software Inc., San Diego, California, USA) and SPSS Statistics 26 (IBM Corporation). A *p* < 0.05 (two‐sided) was considered statistically significant.

## 3 Results

### 3.1 Impact of the β_2_‐Adrenoreceptor Arg16Gly Polymorphism on Asthma Severity

A total of 131 patients with severe eosinophilic asthma treated with mepolizumab were enrolled. Of these, 115 were genotyped for the Arg16Gly polymorphism of the β_2_‐adrenoreceptor, with 102 included in the analysis. A flow diagram of the study participants is shown in Figure S1. To investigate the prevalence of Arg16Gly genotypes, 31 subjects with mild asthma and 20 healthy controls were also included (Figure S1). The alleles were in Hardy–Weinberg equilibrium in both mild asthmatics (*p* = 0.9961) and healthy controls (*p* = 0.9935). Baseline characteristics of all groups are presented in Table S1.

**FIGURE 1:**
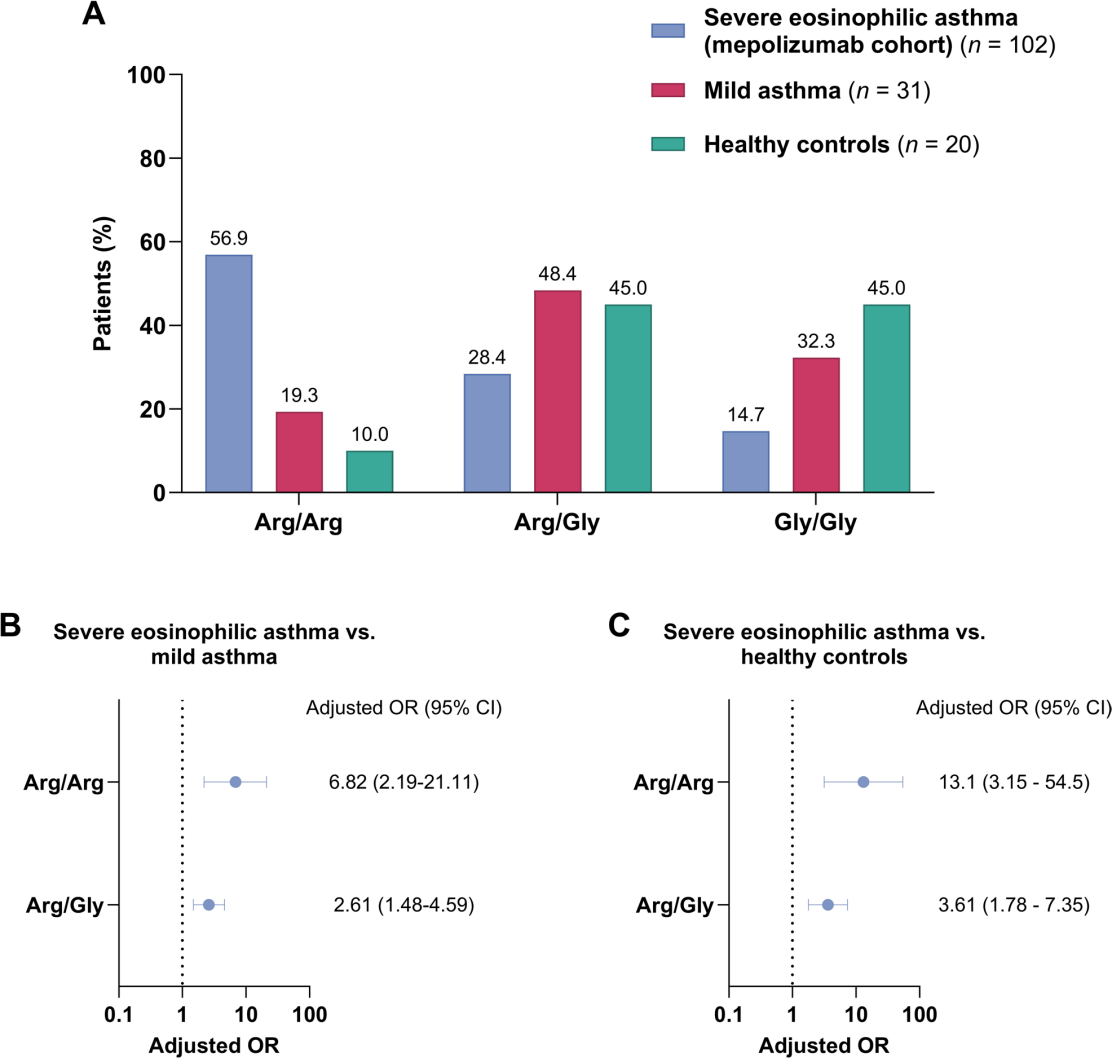
(A) Distribution of β_2_‐adrenoreceptor Arg16Gly genotypes (arginine homozygotes [Arg/Arg], arginine/glycine heterozygotes [Arg/Gly], and glycine homozygotes [Gly/Gly]) in patients with severe eosinophilic asthma (mepolizumab cohort), patients with mild asthma, and healthy controls. (B) Forest plot for the adjusted odds ratios (ORs) and 95% confidence intervals (CIs) of severe eosinophilic asthma compared to mild asthma and (C) healthy controls for each additional Arg16 allele. As an additive allele‐dosing model was used, ORs for the Arg/Arg genotype were derived by squaring the per‐allele OR. The multinomial logistic regression model was adjusted for age, sex, and body mass index (BMI).

The distribution of β_2_‐adrenoreceptor Arg16Gly genotypes in patients with severe eosinophilic asthma (mepolizumab cohort), patients with mild asthma, and healthy controls is presented in Figure 1A. In the severe eosinophilic asthma cohort, Arg/Arg was the most prevalent genotype (56.9%), while Gly/Gly accounted for 14.7%. In contrast, among mild asthmatics, Arg/Arg was found in 19.4% and Gly/Gly in 32.3%. Similarly, in the healthy control group, Arg/Arg accounted for 10% and Gly/Gly for 45%. Multinomial logistic regression analysis revealed that each additional Arg16 allele significantly increased the odds of severe eosinophilic asthma compared with mild asthma (adjusted OR per allele increase: 2.61 [95% CI 1.48–4.59]; *p* = 0.0001) (Figure 1B). Similarly, compared with healthy controls, each additional Arg16 allele was associated with significantly greater odds of severe eosinophilic asthma (adjusted OR per allele increase: 3.61 [95% CI 1.78–7.35]; *p* < 0.0001) (Figure 1C).

### 3.2 Baseline Characteristics of the Mepolizumab Cohort

The baseline characteristics of the mepolizumab cohort are presented in Table 1. The majority of patients were female (69 of 102, 67.3%), with a mean age of 56.5 ± 11.5 years and a median age at onset of 35 (25–49) years. All patients were on high‐dose ICS plus LABA and used SABA as needed; 78.4% were on OCS. No patients received prior treatments with other biologics. There were no statistically significant differences in baseline demographic and clinical characteristics across the Arg/Arg, Arg/Gly, and Gly/Gly genotypes.

**TABLE 1:** Baseline characteristics of the severe eosinophilic asthma (mepolizumab) cohort across Arg16Gly genotypes.

	All (*n* = 102)	Arg/Arg (*n* = 58)	Arg/Gly (*n* = 29)	Gly/Gly (*n* = 15)	*p*
Female, *n* (%)	69 (67.6)	38 (65.5)	22 (75.9)	9 (60)	0.9797
BMI, mean (SD)	27 (5.8)	26.9 (6.5)	27.9 (5.5)	25.7 (3.1)	0.4872
Age, years, mean (SD)	56.5 (11.5)	56 (11)	60 (10)	52 (13.4)	0.0809
Age at onset, years, median (IQR)	35 (25–49)	34 (25.3–48.8)	42 (25–50)	33 (23–44)	0.6218
Age at onset < 18 years, *n* (%)	13 (12.7)	7 (12.1)	4 (13.8)	2 (13.3)	0.8458
Patients with positive Skin Prick Tests, *n* (%)	61 (59.8)	34 (58.6)	18 (62.1)	9 (60)	0.8438
Patients with positive Skin Prick Tests (perennial aeroallergens), *n* (%)	34 (33.3)	16 (27.6)	11 (37.9)	7 (46.7)	0.1267
Race					
White, *n* (%)	102 (100)	58 (100)	29 (100)	15 (100)	0.9999
Smoking status					
Smoking history, *n* (%)	30 (29.2)	16 (27.6)	10 (34.6)	4 (26.7)	0.8480
Current smoker, *n* (%)	6 (5.9)	2 (3.4)	2 (6.9)	2 (13.3)	0.1467
Comorbidities					
Patients with GERD, *n* (%)	38 (37.3)	21 (36.2)	10 (34.5)	7 (46.7)	0.5728
Patients with bronchiectasis, *n* (%)	32 (31.4)	19 (32.8)	10 (34.5)	3 (20)	0.4653
Patients with AERD, *n* (%)	5 (4.9)	2 (3.4)	2 (6.9)	1 (6.7)	0.5407
Patients with CRSwNP, *n* (%)	58 (56.9)	34 (58.6)	18 (62.4)	6 (40)	0.3334
Asthma outcomes					
Exacerbations/year, median (IQR)	5 (4–8)	5 (3–6.8)	6 (4–9.8)	5 (4–6)	0.5217
Patients who required ER/hospitalization, *n* (%)	34 (33.3)	19 (32.8)	10 (34.5)	5 (33.3)	0.9240
ACT, median (IQR)	13 (10–17)	13 (9.8–18)	13.5 (9–17.8)	12 (10–15)	0.8360
FEV_1_, %, mean (SD)	72 (20.7)	71.7 (21)	72.7 (22.9)	71.6 (15.3)	0.9790
FEV_1_, L, mean (SD)	1.87 (0.76)	1.87 (0.8)	1.86 (0.8)	1.89 (0.5)	0.9910
FVC, %, mean (SD)	92 (20.6)	90 (20.7)	91 (19)	93 (23)	0.9860
FEV_1_/FVC, %, mean (SD)	66 (16)	66.5 (15.3)	66 (19)	64 (11)	0.8567
FEF_25%–75%_, %, mean (SD)	43.8 (22.9)	43 (21)	45 (28)	43 (21)	0.9440
Pharmacologic therapies					
High dose ICS‐LABA, *n* (%)	102 (100)	58 (100)	29 (100)	15 (100)	0.9999
LAMA, *n* (%)	68 (66.7)	38 (65.5)	20 (69)	10 (66.7)	0.8486
As‐needed SABA, *n* (%)	49 (48)	28 (48.3)	14 (48.3)	7 (46.7)	0.9261
Patients on OCS, *n*, (%)	80 (78.4)	47 (81)	22 (75.9)	11 (73.3)	0.4553
OCS, mg/die, median (IQR)	12.5 (5–25)	12.5 (5–25)	12.5 (2.5–25)	10 (0–25)	0.3142
LTRA, *n* (%)	59 (48)	26 (44.8)	12 (41.4)	11 (73.3)	0.1264
Previous anti‐IgE/anti‐IL‐5Rα/anti‐IL‐4Rα mAbs, *n* (%)	0 (0)	0 (0)	0 (0)	0 (0)	0.9999
Biomarkers					
Blood eosinophils, cells/μL median (IQR)	596 (420–990)	545 (440–985)	655 (405–1249)	580 (400–780)	0.6330
Blood basophils, cells/μL median (IQR)	50 (30–80)	50 (30–90)	55 (29.8–128)	45 (40–57.5)	0.9199
IgE, UI/mL, median (IQR)	166 (73–315)	165 (74–376)	106 (36.3–254)	186 (139–331)	0.2678
FeNO, ppb, median (IQR)	37 (14–63)	34.5 (12–60)	49 (23.5–84)	34.5 (13.8–51.3)	0.2194

*Note:* Continuous variables are reported as mean and standard deviation (SD) if normally distributed, or as median and interquartile range (IQR) if non‐normally distributed. Categorical variables are presented as numbers (*n*) and percentages (%). For comparisons of continuous variables, ANOVA or the Kruskal–Wallis test was applied, when appropriate. The Cochran‐Armitage test for trend was used for categorical variables.

Abbreviations: ACT, Asthma Control Test; BMI, body mass index; FEF_25%–75%_, forced expiratory flow between 25% and 75% of FVC; FeNO, fractional exhaled nitric oxide; FEV_1_, forced expiratory volume in one second; FVC, forced vital capacity; GERD, gastro‐esophageal reflux disease; ICS‐LABA, inhaled corticosteroids—long‐acting β_2_‐agonist; IgE, immunoglobulin‐E; LAMA, long‐acting muscarinic antagonist; LTRA, Leukotriene receptor antagonists; mAb, monoclonal antibody; OCS, oral corticosteroids (prednisone equivalent dose); SABA, short‐acting β_2_‐agonist.

### 3.3 Long‐Term Mepolizumab Effectiveness According to Arg16Gly Polymorphism Genotypes

**FIGURE 2:**
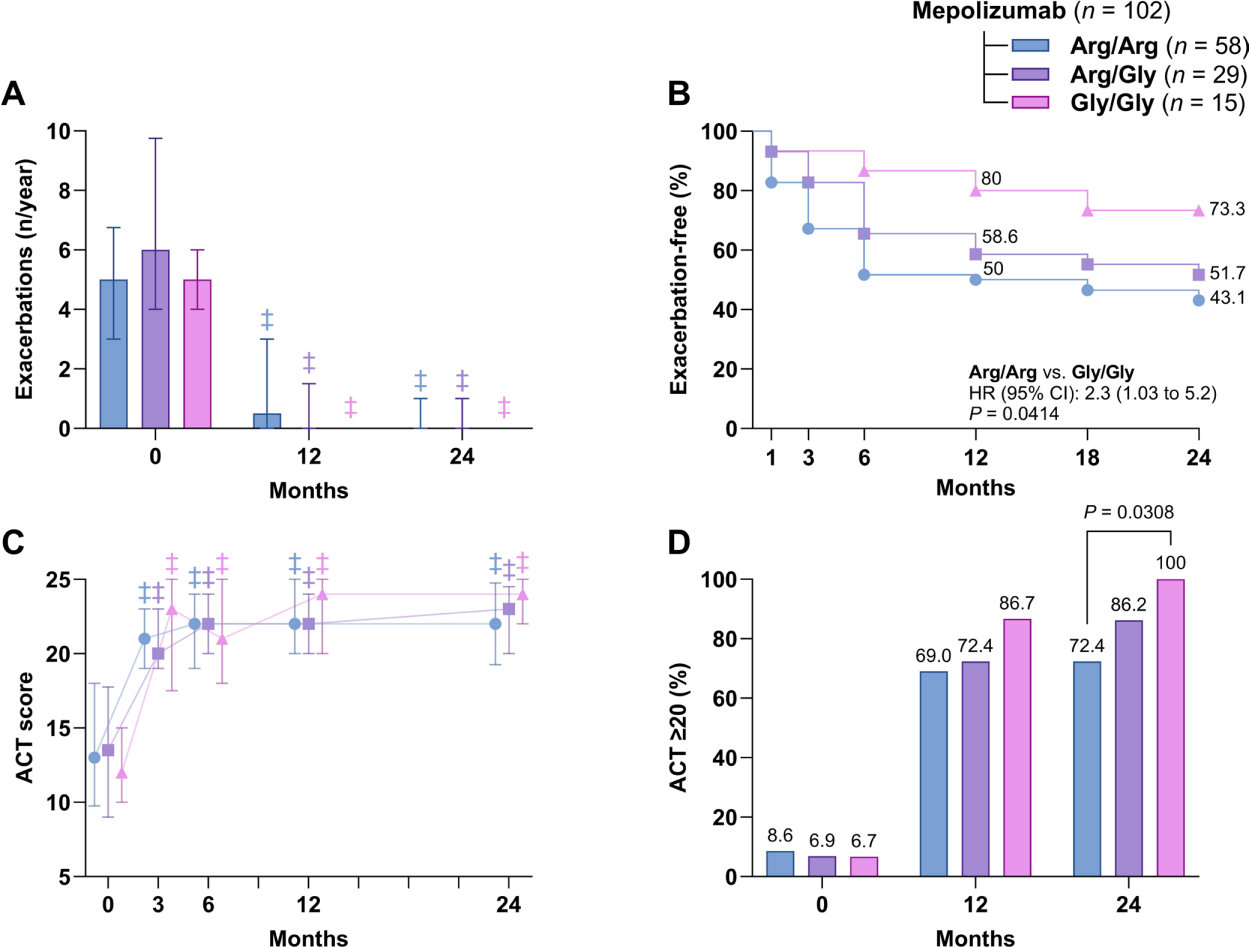
Effectiveness of mepolizumab over 24 months, stratified by β_2_‐adrenergic receptor Arg16Gly polymorphism genotypes (arginine homozygotes [Arg/Arg], arginine/glycine heterozygotes [Arg/Gly], and glycine homozygotes [Gly/Gly]). (A) Annual exacerbation rate (severe exacerbations per year) at baseline (0), 12 months, and 24 months. (B) Kaplan–Meier curves of the proportion of patients remaining free of exacerbations over 24 months, with the hazard ratio (HR) and 95% confidence interval (CI) comparing Arg/Arg versus Gly/Gly. (C) Asthma Control Test (ACT) score at baseline, 3, 6, 12, and 24 months. (D) Proportion of patients achieving well‐controlled asthma (ACT ≥ 20) at baseline, 12 months, and 24 months. *p*‐values for statistically significant between‐genotype comparisons are shown (Fisher exact test). Data are presented as median and interquartile range for the error bars or as percentages of the total. Comparisons of continuous outcomes from 3 to 24 months versus baseline were performed using the Friedman test followed by Dunn's post hoc test. Symbols indicate statistically significant within‐group changes from baseline (^‡^ *p* < 0.001).

The median annual exacerbation rate decreased significantly after 12 and 24 months (*p* < 0.0001 for each genotype vs. baseline) (Figure 2A, Table 2). However, patients with the Arg/Arg genotype were more likely to experience exacerbations at any time during the study compared to Gly/Gly patients (HR 2.3 [95% CI 1.03–5.2]; *p* = 0.0414) (Figure 2B).

**TABLE 2:** Outcomes changes after 12 and 24 months of treatment.

Asthma outcomes	12 months				24 months			
	Arg/Arg (*n* = 58)	Arg/Gly (*n* = 29)	Gly/Gly (*n* = 15)	*p*	Arg/Arg (*n* = 58)	Arg/Gly (*n* = 29)	Gly/Gly (*n* = 15)	*p*
Exacerbations/year, median (IQR)	0.5 (0–3)	0 (0–1.5)	0 (0–0)	—	0 (0–1)	0 (0–1)	0 (0–0)	—
Exacerbations/year, change from baseline, median (95% CI)	−4.5 (−3 to −4)	−6 (−4 to −6)	−5 (−4 to −6)	**0.0450** ^a^	−5 (−4 to −5)	−6 (−4 to −6)	−5 (−4 to −6)	0.1490
ACT, median (IQR)	22 (20–25)	22 (20–24)	24 (20–25)	—	22 (19.3–24.8)	23 (20–24.5)	24 (22–25)	—
ACT, change from baseline, median (95% CI)	+9 (+6 to +10)	+8.5 (+6 to +11)	+12 (+8 to +13)	0.3260	+9 (+6 to +10)	+9.5 (+6 to +11)	+12 (+9 to +13)	0.2680
ACT MCID, *n* (%)	41 (70.7)	24 (85.7)	13 (86.7)	0.1202	47 (82.8)	26 (89.7)	15 (100)	0.1544
ACT ≥ 20, *n* (%)	40 (69)	21 (72.4)	13 (86.7)	0.0866	42 (72.4)	25 (86.2)	15 (100)	**0.0330** ^b^
FEV_1_, %, mean (SD)	82.6 (21)	84.9 (16.9)	85.6 (18.2)	—	80.4 (24.8)	82.3 (18.9)	87.2 (21.5)	—
FEV_1_, %, change from baseline, mean (95% CI)	+11 (+4.4 to +17)	+12.1 (+1 to +23)	+13.9 (+8 to +19)	0.7970	+8.6 (+2.4 to +15)	+9.6 (−2 to +21)	+15.5 (+5 to +25.8)	0.7870
FEV_1_ ≥ 80%, *n* (%)	30 (51.7)	17 (58.6)	10 (66.7)	0.2733	28 (48.3)	14 (48.3)	10 (66.7)	0.2896
FEV_1_ decline ≤ 5% from baseline, *n* (%)	49 (84.5)	25 (86.2)	14 (93.3)	0.4105	49 (84.5)	25 (86.2)	14 (93.3)	0.8501
FEV_1_, L, mean (SD)	2.2 (0.83)	2.1 (0.6)	2.3 (0.5)	—	2.1 (0.6)	2 (0.6)	2.3 (0.6)	—
FEV_1_, L, change from baseline, mean (95% CI)	+0.33 (+0.15 to +0.51)	+0.24 (−0.02 to +0.50)	+0.4 (+0.01 to +0.8)	0.8140	+0.2 (−0.01 to +0.4)	+0.18 (−0.01 to +0.5)	+0.45 (+0.05 to +0.8)	0.7340
FEV_1_ + 100 mL, *n* (%)	38 (65.5)	19 (65.5)	11 (73.3)	0.6332	39 (67.2)	19 (65.5)	12 (80)	0.4653
FEV_1_ decline < 100 mL from the best value of the first 12 months, *n* (%)	—	—	—	—	25 (43.1)	10 (34.5)	12 (80)	0.0650^c^
FEV_1_/FVC, %, mean (SD)	68 (13)	69.7 (14.5)	72 (13.4)	—	67.5 (15.4)	72.4 (11.6)	72.7 (11.2)	—
FEV_1_/FVC, %, change from baseline, mean (95% CI)	+1.7 (−3.7 to +7.2)	+4 (−8 to +15)	+7.6 (−3.2 to +18)	0.3160	+1 (−3.3 to +5)	+6.7 (−3 to +16)	+8.2 (−2.4 to +19)	0.3790
FEF_25%–75%_, %, mean (SD)	52 (24)	47 (25)	53.6 (23.8)	—	49.4 (21)	54 (29)	59 (33)	—
FEF_25%–75%_, %, change from baseline, mean (95% CI)	+8.8 (+2 to +16)	+1 (−9 to +11)	+11 (−4.3 to +26.2)	0.6230	+6 (−3.2 to +15)	+8 (−11 to +28)	+16 (−6 to +39)	0.7150
Pharmacologic therapies								
As‐needed SABA, *n* (%)	3 (5.2)	1 (3.4)	2 (13.3)	0.4131	2 (3.4)	1 (3.4)	1 (6.7)	0.7894
SABA withdrawal, *n* (%)	25/28 (89.3)	13/14 (92.9)	5/7 (71.3)	0.3904	26/28 (92.9)	13/14 (92.9)	6/7 (85.7)	0.7835
Patients on OCS, *n* (%)	20 (34.5)	9 (31)	2 (13.3)	0.1478	10 (17.2)	2 (6.9)	0 (0)	0.1280
OCS withdrawal, *n* (%)	27/47 (57.4)	13/22 (59.1)	8/10 (80)	0.2577	37/47 (78.7)	20/22 (90.9)	10/10 (100)	0.2018
OCS, mg/die, median (IQR)	0 (0–5)	0 (0–4.3)	0 (0–0)	—	0 (0–5.8)	0 (0–1)	0 (0–0)	—
OCS, change from baseline, mg/die, median (95% CI)	−12.5 (−5 to −15)	−12.5 (−2.5 to −20)	−10 (−0.0 to −25)	0.5630	−12.5 (−5 to −19)	−12.5 (−2.5 to −25)	−10 (−2.5 to −25)	0.9540
Biomarkers								
Blood eosinophils, cells/μL, median (IQR)	70 (40–109.5)	85 (52.5–210)	60 (43–100)	—	70 (50–112.5)	70 (49.5–97.5)	87.5 (51–130)	—
Blood eosinophils, cells/μL, change from baseline, median (95% CI)	−475 (−406 to −642)	−565 (−355 to −661)	−510 (−35 to −590)	0.1170	−475 (−387 to −695)	−580 (−360 to −910)	−483 (−260 to −705)	0.2160
FeNO, ppb, median (IQR)	23 (11–50)	32 (17.3–56)	26.5 (13.8–46.3)	—	23 (11–47)	21 (11–34)	19 (16.5–39)	—
FeNO, ppb, change from baseline, median (95% CI)	−11.5 (−15 to +6)	−17 (−31 to +10)	−8 (−14 to +5)	0.3540	−11.5 (−16 to +6)	−28 (−43 to +11)	−15.5 (−23 to +8)	0.3210

*Note:* Continuous variables are reported as mean and standard deviation (SD) if normally distributed, or as median and interquartile range (IQR) if non‐normally distributed. Changes from baseline are presented as mean or median with 95% confidence intervals (CIs). Categorical variables are presented as counts (*n*) and percentages (%). A linear mixed‐effects model—with patients as a random effect and genotypes, age, and time as fixed effects—was used to compare treatment response in parametric variables; Bonferroni post hoc was applied for multiple comparisons. The Jonckheere–Terpstra test, also with Bonferroni post hoc, was used for nonparametric variables. The Cochran‐Armitage test for trend or the Fisher exact test, with or without Freeman–Halton extension, were used for comparisons of categorical variables. Bold indicates statistically significant *p*‐values.

Abbreviations: ACT, Asthma Control Test; FEF_25%–75%_, forced expiratory flow between 25% and 75% of FVC; FeNO, fractional exhaled nitric oxide; FEV_1_, forced expiratory volume in one second; FVC, forced vital capacity; MCID, minimal clinically important difference; OCS, oral corticosteroids (prednisone equivalent dose); SABA, short‐acting β_2_‐agonist.

^a^ A Jonckheere–Terpstra test revealed a significant overall trend across the ordered genotypes. However, pairwise comparisons among the three groups did not remain significant after Bonferroni correction.

^b^ Fisher exact test with Freeman–Halton extension; *p* = 0.0308 for Arg/Arg versus Gly/Gly (Fisher exact test).

^c^ Cochran‐Armitage test for trend; *p* = 0.0097 for Arg/Gly versus Gly/Gly (Fisher exact test), *p* = 0.0186 for Arg/Arg versus Gly/Gly (Fisher exact test).

Regarding asthma symptoms, the ACT score increased to a median of 20 already after 3 months (*p* < 0.0001 for each genotype at every time point vs. baseline) (Figure 2C). After 24 months, the proportion of patients with an ACT score ≥ 20 was significantly higher in the Gly/Gly genotype (100%) than in Arg/Arg (72.4%, *p* = 0.0308) (Figure 2D).

**FIGURE 3:**
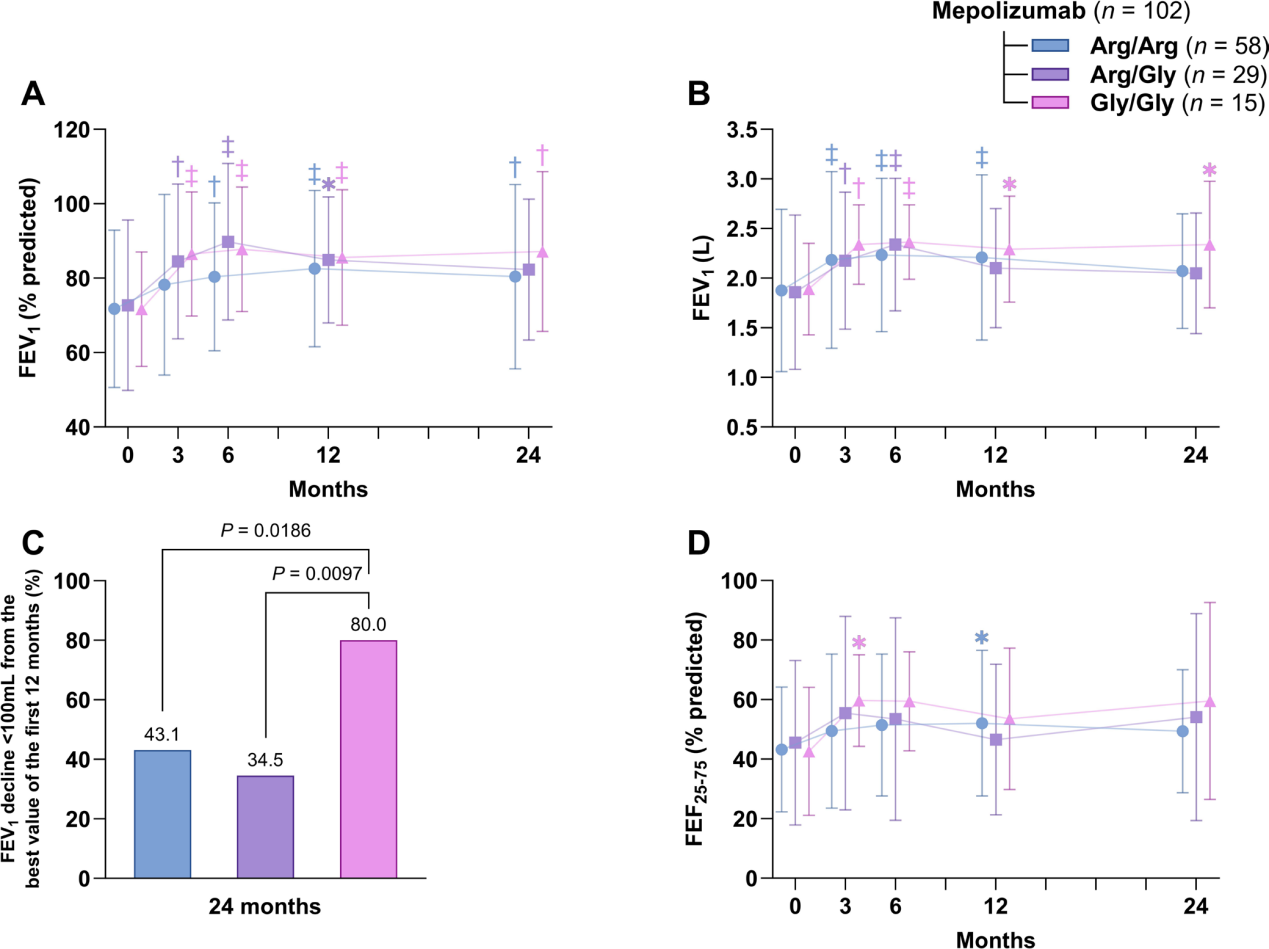
Impact of mepolizumab over 24 months, stratified by β_2_‐adrenergic receptor Arg16Gly polymorphism genotypes (arginine homozygotes [Arg/Arg], arginine/glycine heterozygotes [Arg/Gly], and glycine homozygotes [Gly/Gly]) on lung function parameters. (A) Pre‐bronchodilator forced expiratory volume in one second (FEV_1_) as a percentage of the predicted value. (B) Pre‐bronchodilator FEV_1_ (L). (C) Proportion of patients whose FEV_1_ declined by less than 100 mL from the best value recorded in the first 12 months, assessed at 24 months. *p*‐values for statistically significant between‐genotype comparisons are shown (Fisher exact test). (D) Pre‐bronchodilator forced expiratory flow between 25% and 75% of FVC (FEF_25%–75%_) expressed as a percentage of the predicted value. Data are presented as mean and standard deviation for the error bars or as percentages of the total. Comparisons of continuous outcomes from 3 to 24 months versus baseline were performed using Dunnett's test. Symbols indicate statistically significant within‐group changes from baseline (*p < 0.05; ^†^ *p* < 0.01; ^‡^ *p* < 0.001).

During the first 6 months, both pre‐bronchodilator FEV_1_% and FEV_1_ (L) significantly increased (Figure 3A,B). At month 12, we noted better improvements in Gly/Gly in comparison to Arg/Arg and Arg/Gly, although between‐groups differences were not statistically significant (Table 2). A slight decline in both FEV_1_% and FEV_1_ (L) was observed after the first year, which was less pronounced in Gly/Gly patients. Indeed, 80% of Gly/Gly patients had an FEV_1_ decline < 100 mL from the best value in the first 12 months, compared to 43.1% of Arg/Arg (*p* = 0.0108) and 34.5% of Arg/Gly (*p* = 0.0042) (Figure 3C). The FEV_1_/FVC% significantly improved only in the Gly/Gly group after 6 months and remained stable thereafter (Figure S2). A similar trend was observed for the FEF_25%–75%_ (Figure 3D).

**FIGURE 4:**
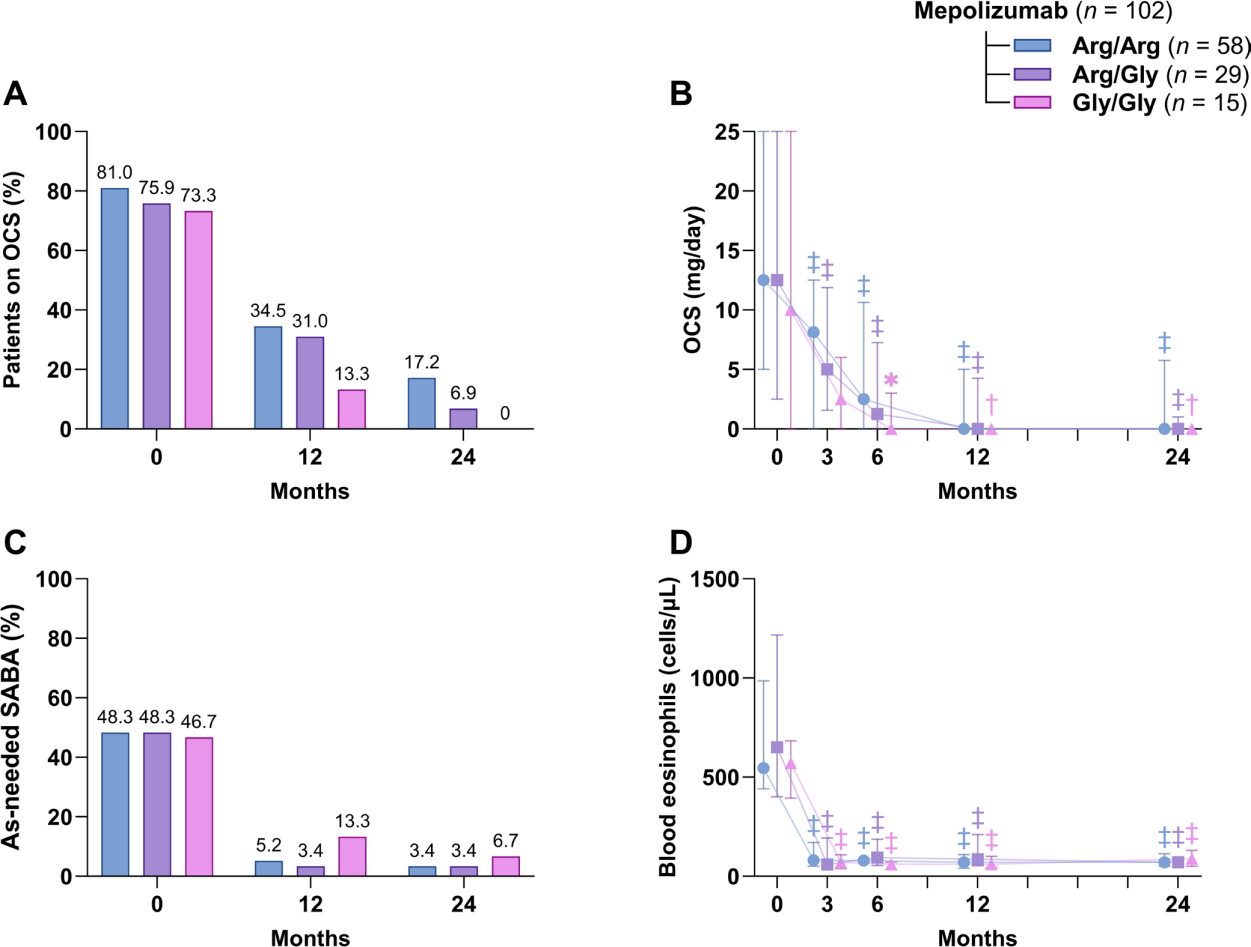
Changes in (A) percentage of patients on oral corticosteroids (OCS), (B) daily OCS dose (mg/day, prednisone equivalent dose), (C) percentage of patients using as‐needed short‐acting β_2_‐agonist (SABA), and (D) blood eosinophil counts (cells/μL) stratified by β_2_‐adrenergic receptor Arg16Gly polymorphism genotypes (arginine homozygotes [Arg/Arg], arginine/glycine heterozygotes [Arg/Gly], and glycine homozygotes [Gly/Gly]). Data are presented as median and interquartile range for the error bars or as percentages of the total. Comparisons of continuous outcomes from 3 to 24 months versus baseline were performed using the Friedman test followed by Dunn's post hoc test. Symbols indicate statistically significant within‐group changes from baseline (*p < 0.05; ^†^ *p* < 0.01; ^‡^ *p* < 0.001).

After 24 months, only 17.2% Arg/Arg and 6.9% Arg/Gly remained on OCS (Figure 4A). No Gly/Gly patients required OCS after 2 years (Table 2, Figure 4A). The median OCS dose reached zero by month 12 for all genotypes (Figure 4B). The percentage of patients requiring as‐needed SABA declined over time (Figure 4C), with no differences between genotypes after 12 and 24 months of treatment (Table 2). Mepolizumab treatment led to a significant drop in peripheral blood eosinophil count (*p* < 0.0001 for each genotype at every timepoint vs. baseline), and this reduction persisted throughout the study (Figure 4D).

### 3.4 Clinical and Sustained Remission According to Arg16Gly Polymorphism Genotypes

**FIGURE 5:**
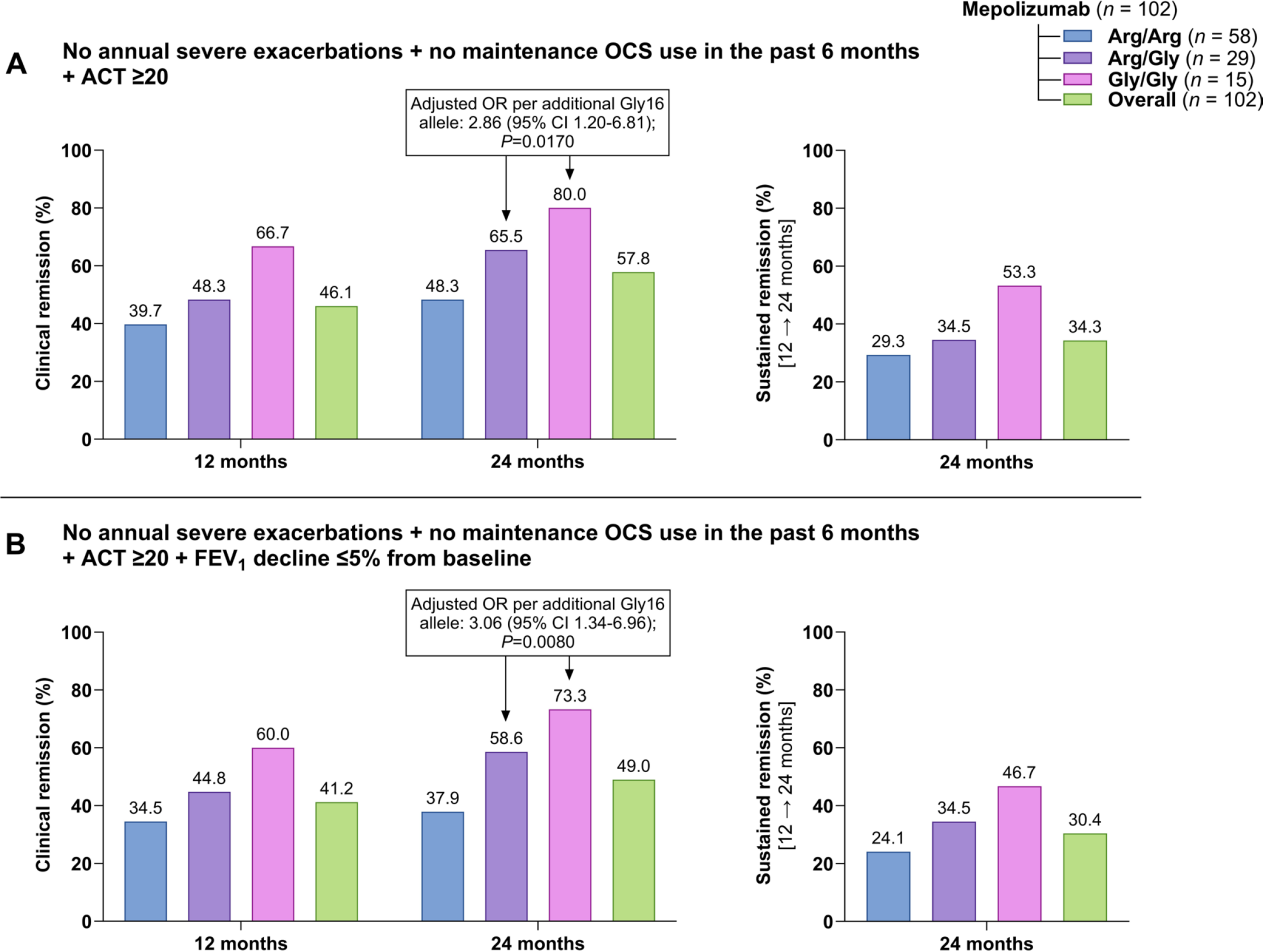
Rates of clinical and sustained remission according to the (A) *main definition* (3‐component) and (B) *secondary definition* (4‐component) after 12 and 24 months of mepolizumab treatment, stratified by β_2_‐adrenergic receptor Arg16Gly genotype (arginine homozygotes [Arg/Arg], arginine/glycine heterozygotes [Arg/Gly], and glycine homozygotes [Gly/Gly]) and for the overall cohort. Data are presented as percentages of the total. Adjusted odds ratios (ORs) were estimated using binary logistic regression (with the clinical remission definitions as the dependent variables). Genotype was included as a covariate, and the models were adjusted for study center, age, sex, BMI, smoking history, baseline annual exacerbation rate, inhaled therapy, OCS use, leukotriene receptor antagonist therapy, and blood eosinophil count.

Given that Arg/Arg homozygosity is highly prevalent in severe eosinophilic asthma and is associated with β_2_‐receptor downregulation, we aimed to investigate whether the Gly16 allele confers a protective effect and identifies a subset of patients who exhibit a higher likelihood of clinical remission. The proportion of patients who achieved clinical remission differed according to the definition applied and across genotypes (Figure 5, Figure S3). Secondary failure rates, defined as achieving clinical remission at 12 months and subsequently losing remission status at later follow‐up visits, are presented in Table S2.

Applying the *main definition* (3‐component) of remission (Figure 5A), remission rates at 12 months progressively increased with each additional Gly16 allele; however, the Gly16 allele was not identified as an independent predictor in the logistic regression analysis. Instead, baseline use of as‐needed SABA markedly reduced the odds of remission (adjusted OR 0.23 [95% CI 0.07–0.80]; *p* = 0.0210), whereas a higher blood eosinophil count slightly increased the likelihood (adjusted OR 1.002 [95% CI 1.000–1.003]; *p* = 0.0220) (Table S3). By 24 months, 48.3% of Arg/Arg, 65.5% of Arg/Gly, and 80% of Gly/Gly patients met the main remission criteria (Figure 5A). Logistic regression showed that each additional Gly16 allele nearly tripled the odds of remission (adjusted OR 2.86 [95% CI 1.20–6.81]; *p* = 0.0170) (Table S3). Sustained remission through 24 months remained negatively associated with baseline SABA use and positively associated with blood eosinophil count (Table S3). The Gly16 allele was not an independent predictor of sustained remission.

With the *secondary definition* (4‐component), baseline blood‐eosinophil count was the sole independent predictor of remission at 12 months (adjusted OR 1.001 [95% CI 1.000–1.003]; *p* = 0.0360) (Table S4). At 24 months, remission was achieved by 37.9% of Arg/Arg, 58.6% of Arg/Gly, and 73.3% of Gly/Gly patients (Figure 5B), and in the logistic regression model the Gly16 allele was the only significant predictor (adjusted OR 3.06 [95% CI 1.34–6.96]; *p* = 0.0080) (Table S4). Baseline SABA use was negatively associated with achieving sustained remission at 24 months (Table S4).

## 4 Discussion

In this study, we found a higher prevalence of the Arg16 allele (Arg16Gly, rs1042713) in patients with severe eosinophilic asthma than in patients with mild asthma or healthy controls. Each additional Arg16 allele tripled the odds of severe eosinophilic asthma relative to mild asthma and quadrupled it relative to healthy controls. Our findings also suggest that this polymorphism significantly impacts the long‐term effectiveness of mepolizumab. Patients with the Arg/Arg genotype had a higher risk of exacerbations, poorer asthma control, and steeper lung‐function decline than Gly/Gly homozygotes. In contrast, each additional Gly16 allele tripled the odds of achieving clinical remission after 24 months under both the *main* (3‐component) and *secondary* (4‐component) definitions.

The β_2_‐adrenergic receptor is a G‐protein‐coupled receptor encoded by a gene located on chromosome 5 (5q32). It exerts multiple effects in the airways, including smooth muscle relaxation, inhibition of acetylcholine release, increased ciliary beat frequency, reduced venular permeability, and suppression of pro‐inflammatory mediator release from immune cells, thereby contributing to an overall anti‐inflammatory effect [34]. The rs1042713 polymorphism results from a single nucleotide substitution at position 46 (A → G), encoding an arginine instead of a glycine at position 16. This substitution enhances agonist‐promoted receptor downregulation and uncoupling, leading to tachyphylaxis [20].

Overall, we found that the prevalence of the Arg16 allele exceeds 85% in patients with severe eosinophilic asthma. Since the β_2_‐adrenoreceptor inhibits the release of pro‐inflammatory molecules from ILC2, Th‐2 lymphocytes, mast cells, macrophages, and eosinophils and limits their recruitment, increased receptor downregulation may contribute to a pro‐inflammatory imbalance [34, 47]. Nevertheless, the observation that some healthy individuals carry the Arg16 allele without developing asthma underlies that genetic predisposition alone is insufficient, suggesting that additional environmental exposures, gene–gene interactions, or epigenetic factors likely contribute to asthma manifestation and severity.

Our findings suggest that patients carrying the Arg/Arg genotype are more likely to experience exacerbations and suboptimal asthma control during 24 months of mepolizumab treatment compared to those with the Gly/Gly genotype. However, the exacerbation rate in Arg/Arg individuals was consistent with those reported in clinical trials and real‐world studies [1, 2, 3, 4, 5, 6], likely reflecting the high prevalence of this genotype among patients with severe asthma.

The Arg/Arg genotype has been associated with an altered response to SABA and reduced bronchoprotection with LABA, even when combined with ICS [26, 27, 28, 29, 30, 31, 32, 48, 49, 50, 51]. Clinically, reduced bronchoprotection directly impacts airway smooth muscle stabilization and airway geometry. Furthermore, β_2_‐agonists normally inhibit airway smooth muscle mitogenesis [52] as well as the expression of key eosinophil‐attracting chemokines, such as eotaxin [53] and RANTES [54]. In individuals with the Arg/Arg genotype, blunted β_2_‐adrenergic activity may not only diminish its inhibitory effects on the release of pro‐inflammatory mediators from Th2 lymphocytes, mast cells, and eosinophils [34, 47]—thereby enhancing their trafficking [34]—but may also foster increased expression of eotaxin and RANTES. Additionally, β_2_ receptor signaling has been shown to limit the activation of ILC2 [33], a predominant source of type 2 cytokines in corticosteroid‐resistant severe eosinophilic asthma [55, 56]. Collectively, these mechanisms could predispose Arg/Arg genotype carriers to heightened susceptibility to eosinophilic exacerbations, corticosteroid resistance, persistent symptoms, and accelerated lung function decline [57, 58, 59]. Notably, mepolizumab at the standard 100 mg dose is only partially effective at controlling IL‐5‐driven sputum eosinophilia and reducing ILC2 numbers [60, 61, 62, 63, 64]. To the best of our knowledge, no studies have directly compared IL‐5, eotaxin, or blood/sputum eosinophil levels between Arg/Arg and Gly/Gly carriers. Future research should therefore clarify whether the rs1042713 polymorphism, particularly the Arg/Arg genotype, is associated with a more pronounced airway eosinophilic inflammation and airway remodeling. We observed a nonsignificant trend toward a higher prevalence of bronchiectasis among carriers of the Arg16 allele; however, there is currently no evidence explicitly linking the Arg16Gly polymorphism to bronchiectasis risk in severe eosinophilic asthma.

While the percentage of Arg/Arg homozygotes achieving clinical remission aligns with previous mepolizumab studies, Gly/Gly patients showed higher rates of clinical and sustained remission after 24 months. Indeed, each Gly16 allele tripled the odds of achieving clinical remission under both the *main* (3‐component) and *secondary* (4‐component) definitions. Although intrinsic genotype‐driven differences in response to mepolizumab started to manifest within the first 12 months, clinical outcomes such as exacerbations, symptoms, and corticosteroid sparing typically evolve progressively, possibly explaining why significant genotype‐specific differences in clinical remission became apparent only after the first year of treatment.

This study has several limitations. We did not collect genome‐wide genotyping data, which prevented us from inferring genetic ancestry or formally adjusting for population stratification and relatedness. Any unrecognized ancestry differences within our cohort could thus have introduced confounding and potentially led to false‐positive associations. All participants were self‐identified white individuals from Southern Italy. This may limit the generalizability of our findings to other ethnic groups or geographic populations; therefore, the lack of external validity is an additional limitation that should be addressed in future studies. Since the amplicons were not confirmed by direct sequencing or state‐of‐the‐art genotyping methods, genotyping errors cannot be entirely excluded. Genotype‐stratified measurements of airway eosinophils and their activation markers were not performed. The relatively small number of controls may have contributed to a wider 95% CI for the risk estimates associated with carrying the Arg16 allele. We did not include a non‐mepolizumab treated severe asthma group, precluding direct comparisons between treated and untreated patients. Despite robust statistical adjustments for multiple variables known to influence mepolizumab response, we acknowledge the possibility of residual confounding due to unmeasured factors. Lastly, some comparisons did not reach statistical significance, maybe due to the small sample size of certain groups.

Nevertheless, our study has several strengths. We reported, for the first time, the prevalence and impact of a common polymorphism in a large cohort of severe eosinophilic asthma patients undergoing long‐term mepolizumab treatment. Data were collected under rigorous standards across multiple sites within a network known for its expertise in severe asthma management, and our findings reflect real‐world clinical practice beyond the strict inclusion criteria of clinical trials. Furthermore, our 24‐month follow‐up provides comprehensive insights into the long‐term effects of the rs1042713 polymorphism on treatment outcomes, including clinical and sustained remission, using various commonly proposed definitions.

In the current post‐genome‐wide association studies era, integrating genomics with other “*omic*” layers—such as transcriptomics, epigenomics, proteomics, metabolomics, and radiomics—[65, 66] is crucial for achieving a more comprehensive understanding of severe asthma pathobiology and enhancing precision medicine strategies [67, 68] through more accurate prediction of treatment responses, particularly in relation to biologic therapies.

In conclusion, this study highlights the potential impact of the β_2_‐adrenergic receptor rs1042713 polymorphism (Arg16Gly) on the effectiveness of mepolizumab and the likelihood of achieving clinical remission in patients with severe eosinophilic asthma. These findings raise the possibility that genetic screening for β_2_‐adrenergic receptor polymorphisms could serve as a biomarker to identify patients who might require alternative or adjunctive therapeutic approaches to optimize their outcomes. Further research is warranted to validate these findings in larger cohorts and explore the broader implications of this polymorphism with other biologics and its relevance in different ethnic groups to further enhance personalized treatment strategies for patients with severe asthma.

## Author Contributions

Conceptualization: S.N., E.F., N.C., and C.C. Study design: N.C., S.N., and C.C. Patient recruitment and patient management: S.N., R.C., A.P., G.S., C.P., A.M., C.Ca., V.N.Q., I.C., A.S., A.V., G.P., C.V., M.P.F.B., M.D., G.E.C., N.C., and C.C. Database: S.N. and E.F. Samples analysis: E.F. Data analysis: S.N., R.C., and C.C. Manuscript draft: S.N. and C.C. All authors have read and approved the submitted manuscript. All authors reviewed the data and contributed to its interpretation, edited the manuscript, and approved the final submitted version.

## Conflicts of Interest

S.N. has received speaker fees from AstraZeneca, GlaxoSmithKline, and Sanofi as well as honoraria for advisory board participation from AstraZeneca and GlaxoSmithKline. N.C. has received speaker and lecturer fees from AstraZeneca, GlaxoSmithKline, and Sanofi. C.C. has received speaker fees from ResMed, Fisher & Paykel, Sanofi, AstraZeneca, GlaxoSmithKline, and Menarini. All other authors declare no conflicts of interest.

## Supporting information


**Data S1:** all70071‐sup‐0001‐supinfo.pdf.

## Data Availability

The data that support the findings of this study are available from the corresponding author upon reasonable request.

## Southern Italy Network on Severe Asthma Therapy

Collaborators: Rossella Intravaia: Respiratory Medicine Unit, Policlinico “G. Rodolico‐San Marco” University Hospital, Catania, Italy; Morena Porto: Department of Clinical and Experimental Medicine, University of Catania, Catania, Italy; Pietro Impellizzeri: Department of Clinical and Experimental Medicine, University of Catania, Catania, Italy; Department of Clinical and Experimental Medicine, University of Catania, Catania, Italy; Maria Provvidenza Pistorio: Department of Clinical and Experimental Medicine, University of Catania, Catania, Italy; Department of Clinical and Experimental Medicine, University of Catania, Catania, Italy; Fabio Vignera: Department of Clinical and Experimental Medicine, University of Catania, Catania, Italy; Andrea Alessia Nardo: Department of Clinical and Experimental Medicine, University of Catania, Catania, Italy; Concetta Giannì: Department of Clinical and Experimental Medicine, University of Catania, Catania, Italy; Silvano Dragonieri: Department of Translational Biomedicine and Neuroscience, University “Aldo Moro”, Bari, Italy; Flogerta SanaDepartment of Translational Biomedicine and Neuroscience, University “Aldo Moro”, Bari, Italy; Department of Translational Biomedicine and Neuroscience, University “Aldo Moro”, Bari, Italy; Valeria Pezzuto: Department of Translational Biomedicine and Neuroscience, University “Aldo Moro”, Bari, Italy; Santina Ferulli: Division of Allergy and Clinical Immunology, University “Aldo Moro”, Bari, Italy; Massimo Triggiani: Division of Allergy and Clinical Immunology, University of Salerno, Italy; Roberta Parente: Department of Medical and Surgical Sciences, University of Foggia, Italy; Pasquale Tondo: Department of Medical and Surgical Sciences, University of Foggia, Italy; Piera Soccio: Department of Medical and Surgical Sciences, University of Foggia, Italy; Donato Lacedonia: Department of Medical and Surgical Sciences, University of Foggia, Italy; Luigi Ciampo: Department of Medicine, Surgery and Dentistry, University of Salerno, Salerno, Italy; Antonio Franzese: Department of Medicine, Surgery and Dentistry, University of Salerno, Salerno, Italy; Valeria Longobardi: Department of Medicine, Surgery and Dentistry, University of Salerno, Salerno, Italy.
