# Supplementary material for: Effect of the Arg16Gly β2‐Adrenergic Receptor Polymorphism on Long‐Term Mepolizumab Response and Clinical Remission in Severe Eosinophilic Asthma: A Genotype‐Stratified, Multicenter Study
Source: Allergy. 2025 Sep 23;81(3):734–49. doi: 10.1111/all.70071 (PMC12954561; doi:10.1111/all.70071)
Supplement: Supplementary file 1 — Data S1: all70071‐sup‐0001‐supinfo.pdf. [file ALL-81-734-s001.pdf]

**SUPPORTING INFORMATION**

***"*SOUTHERN ITALY NETWORK ON SEVERE ASTHMA THERAPY"**

***Recruiting centers***

1. Respiratory Medicine Unit - Policlinico "G. Rodolico-San Marco" University Hospital, Catania;
2. Respiratory Medicine Unit - A.O.U. Policlinico di Bari "Giovanni XXIII", Bari;
3. Institute of Respiratory Diseases - University Hospital of Foggia, Foggia;
4. Pulmonary Unit - A.O.U "Mater Domini", Catanzaro;
5. Respiratory Medicine Unit - A.O.U. "San Giovanni di Dio and Ruggi d'Aragona", Salerno;
6. Allergology and Clinical Immunology - A.O.U. "San Giovanni di Dio and Ruggi d'Aragona", Salerno;
7. Department of Pneumology - A.O. "Dei Colli", Naples.

**Genotyping of the $\beta_2$-adrenergic receptor Arg16Gly (rs1042713) polymorphism**

Genotyping for the Arg16Gly (rs1042713) polymorphism of the $\beta_2$-adrenergic receptor was performed using a combination of primer-induced restriction site assay and restriction fragment length polymorphism (RFLP) with the NcoI restriction enzyme.

Genomic DNA was extracted using the QIAamp DNA Blood Kit (Qiagen) following the manufacturer's protocol and quantified by ultraviolet spectroscopy on a NanoDrop 2000 (Thermo Scientific). One microgram of genomic DNA was utilized to amplify the specific gene sequence encoding the $\beta_2$-adrenergic receptor according to the method previously described by Martinez *et al.* [1], ensuring that the PCR reaction amplified the region containing codon 16 of the receptor. The PCR primers designed for amplification of the $\beta_2$-adrenergic receptor polymorphic region were:

- 5'-GCCTTCTTGCTGGCACCCCAT-3'
- 5'-CAGACGCTCGAACTTGGCCATG-3'

The bases underlined were specifically modified from the original sequence to create NcoI restriction sites.

PCR was conducted using Platinum Taq DNA Polymerase (Thermo Fisher Scientific) under the following thermal cycling conditions repeated for 35 cycles: initial denaturation at 94°C for 30 seconds, annealing at 60°C for 30 seconds, and extension at 72°C for 1 minute. The PCR product generated was 168 bp in length, with an undigested sample used as a control.

For detection of the Arg16Gly polymorphism (rs1042713), 8 µL of the PCR product was digested with 2 U of NcoI enzyme in Buffer 4 (New England Biolabs, Boston, MA) at 37°C for 2 hours. Restriction fragments were resolved by electrophoresis on a precast 4% agarose gel (Thermo Fisher Scientific).

The recognition sequence for NcoI enzyme is: 5'...CCATGG...3' and 3'...GGTACC...5'. NcoI enzyme cuts 22 bp from the 3'-end of both alleles, and additionally, 18 bp from the 5'-end of the Gly16 allele. Consequently, restriction sites serve as markers indicating the presence or absence of gene polymorphisms: specifically, the presence of the Arg16 allele leads to an alteration in the enzyme recognition sequence, eliminating a cutting site and generating a longer fragment compared to the Gly16 allele, thus resulting in length polymorphisms of the fragments obtained.

The fragment sizes observed after digestion correlate to the genotypes:

1. A single 128-bp fragment indicates a homozygous Gly/Gly genotype.
2. A single 145-bp fragment indicates a homozygous Arg/Arg genotype.
3. Both 128-bp and 145-bp fragments indicate a heterozygous Gly/Arg genotype.

Fifteen samples were re-amplified and re-digested blind by a second operator, showing 100% concordance. Because the restriction site is introduced by the primer, the assay is not vulnerable to unknown variants elsewhere in the amplicon. The accuracy of the genotyping method was previously validated by allele-specific PCR that demonstrated complete concordance with direct sequencing results in prior publications [1-3].

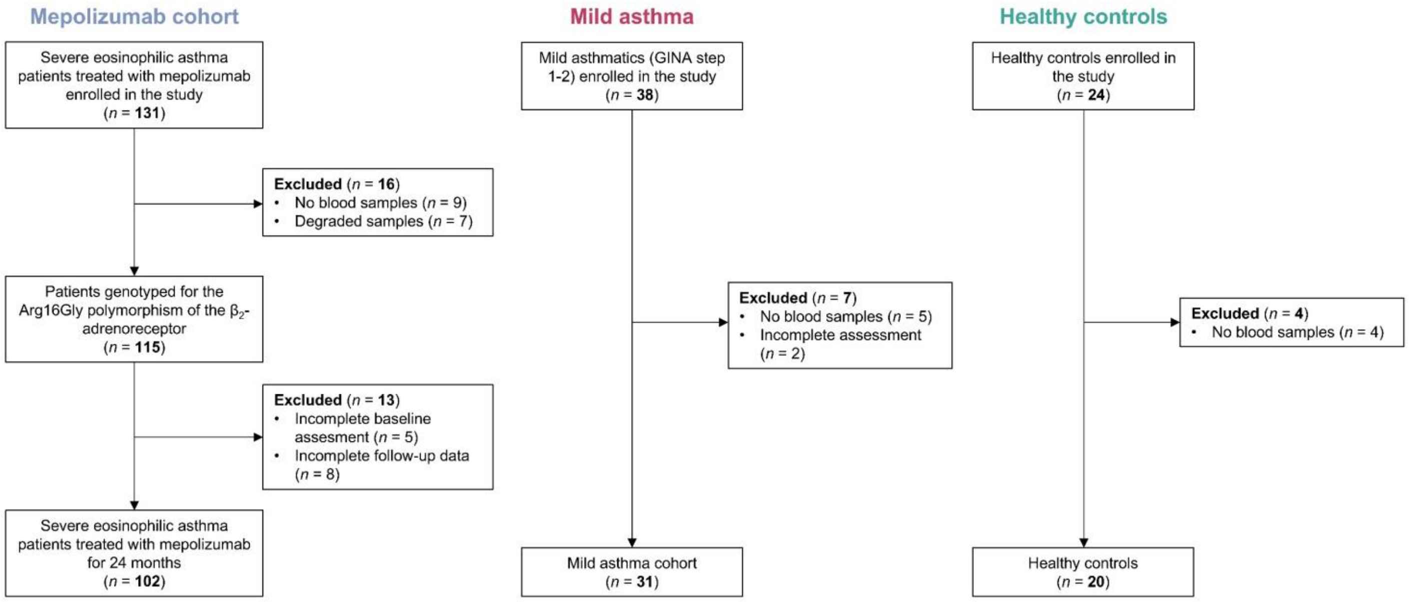


**Figure S1.** Participants flow diagram for each study group.

**Table S1.** Baseline characteristics of severe eosinophilic asthma patients, mild asthmatics and healthy controls

| | **Severe eosinophilic asthma (mepolizumab) (n=102)** | **Mild asthma (n=31)** | **Healthy controls (n=20)** |
|---|---|---|---|
| Female, n (%) | 69 (67.6) | 22 (71) | 13 (65) |
| BMI, mean (SD) | 27 (5.8) | 27.7 (7.8) | 29.3 (8.9) |
| Age, years, mean (SD) | 56.5 (11.5) | 55.3 (12.3) | 55.3 (13.6) |
| Age at onset, years, median (IQR) | 35 (25-49) | 23 (16-31) | - |
| Age at onset <18 years, n (%) | 13 (12.7) | - | - |
| Patients with positive Skin Prick Tests, n (%) | 61 (59.8) | 16 (51.6) | - |
| Patients with positive Skin Prick Tests (perennial aeroallergens), n (%) | 34 (33.3) | 7 (22.6) | - |
| **Race** | | | |
| White, n (%) | 102 (100) | 31 (100) | 20 (100) |
| **Smoking status** | | | |
| Smoking history, n (%) | 30 (29.2) | 6 (19.4) | 4 (20) |
| Current smoker, n (%) | 6 (5.9) | 2 (6.5) | 1 (5) |
| **Comorbidities** | | | |
| Patients with GERD, n (%) | 38 (37.3) | 4 (12.9) | - |
| Patients with bronchiectasis, n (%) | 32 (31.4) | 2 (6.5) | - |
| Patients with AERD, n (%) | 5 (4.9) | 1 (3.2) | - |
| Patients with CRSwNP, n (%) | 58 (56.9) | 3 (9.7) | - |
| **Asthma outcomes** | | | |
| Exacerbations / year, median (IQR) | 5 (4-8) | 0 (0-1) | - |
| Patients who required ER / hospitalization, n (%) | 34 (33.3) | 0 (0) | - |
| ACT, median (IQR) | 13 (10-17) | 25 (22-25) | - |
| $FEV_1$, %, mean (SD) | 72 (20.7) | 95 (18.1) | 101 (17) |
| $FEV_1$, L, mean (SD) | 1.87 (0.76) | 2.5 (0.7) | 2.6 (0.6) |
| FVC, %, mean (SD) | 92 (20.6) | 97 (12.4) | 103 (13) |
| $FEV_1$/FVC, %, mean (SD) | 66 (16) | 81 (10) | 82 (9) |
| $FEF_{25\text{-}75}$, %, mean (SD) | 43.8 (22.9) | 90 (18.2) | 97 (19) |
| **Pharmacologic therapies** | | | |
| High dose ICS-LABA, n (%) | 102 (100) | 0 (0) | - |
| Low dose ICS-LABA, n (%) | 0 (0) | 27 (87.1) | |
| LAMA, n (%) | 68 (66.7) | 0 (0) | - |
| As-needed SABA, n (%) | 49 (48) | 4 (12.9) | - |
| Patients on OCS, n, (%) | 80 (78.4) | 0 (0) | - |
| OCS, mg/die, median (IQR) | 12.5 (5-25) | 0 (0) | - |
| LTRA, n (%) | 59 (48) | 5 (16.1) | - |
| Previous anti-IgE/anti-IL-5Rα/anti-IL-4Rα mAbs, n (%) | 0 (0) | - | - |
| **Biomarkers** | | | |
| Blood eosinophils, cells/µL median (IQR) | 596 (420-990) | 162 (80-310) | 90 (0-180) |
| Blood basophils, cells/µL median (IQR) | 50 (30-80) | 50 (20-80) | 40 (30-90) |
| IgE, UI/ml, median (IQR) | 166 (73-315) | 143 (34-293) | - |
| FeNO, ppb, median (IQR) | 37 (14-63) | 24 (9-36) | 13 (5-27) |

Continuous variables are reported as mean and standard deviation (SD) if normally distributed, or as median and interquartile range (IQR) if non-normally distributed. Categorical variables are presented as numbers (n) and percentages (%).

*Abbreviations: AERD, aspirin exacerbated respiratory disease; ACT, Asthma Control Test; BMI, body mass index;* $FEF_{25\text{-}75}$*, forced expiratory flow between 25% and 75% of FVC; FeNO, fractional*

*exhaled nitric oxide; $FEV_1$, forced expiratory volume in one second; FVC, forced vital capacity; ICS-LABA, inhaled corticosteroids - long-acting $\beta_2$-agonist; IgE, immunoglobulin-E; GERD, gastro-esophageal reflux disease; LAMA, long-acting muscarinic antagonist; LTRA, Leukotriene receptor antagonists; mAb, monoclonal antibody; OCS, oral corticosteroids (prednisone equivalent dose); SABA, short-acting $\beta_2$-agonist.*

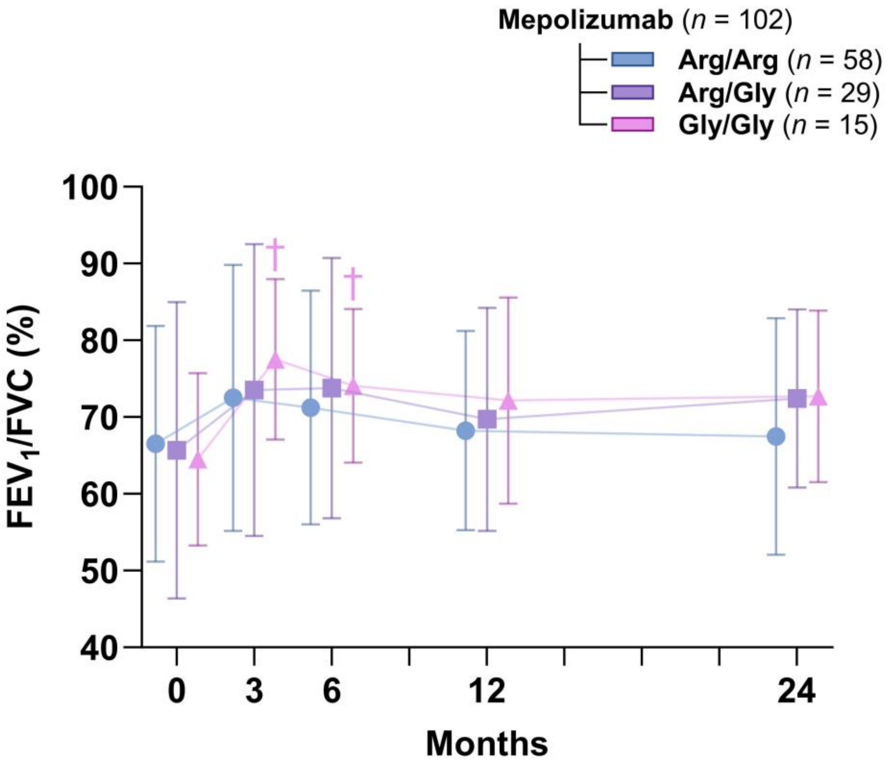


**Figure S2.** Forced expiratory volume one second ($FEV_1$) / forced vital capacity (FVC) ratio during mepolizumab treatment over 24 months, stratified by $\beta_2$-adrenergic receptor Arg16Gly polymorphism genotypes [arginine homozygotes (Arg/Arg), arginine/glycine heterozygotes (Arg/Gly), and glycine homozygotes (Gly/Gly)].

Data are presented as mean and errors bars are standard deviations. Symbols indicate statistically significant within-group changes from baseline (†P<0.01).

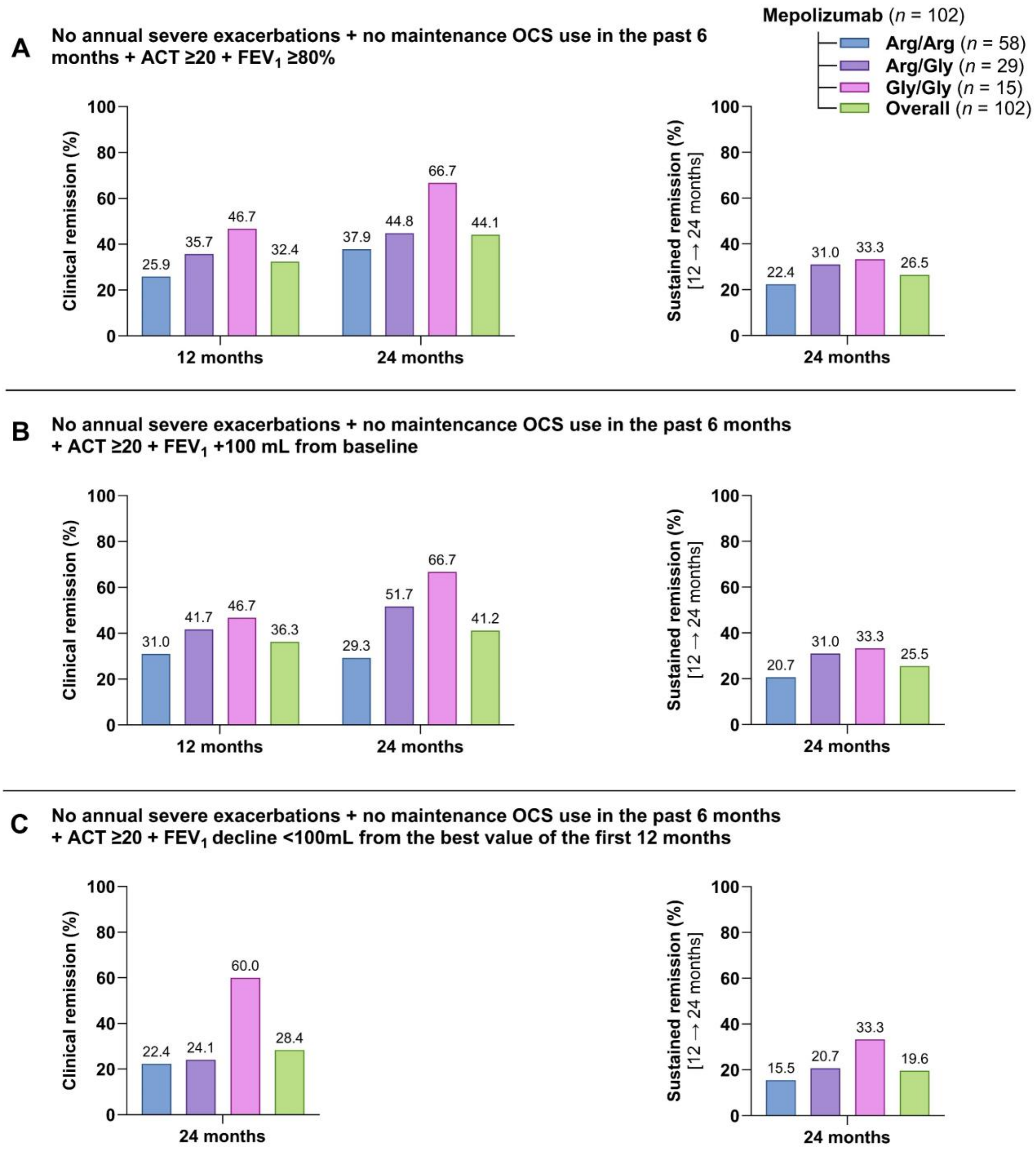


**Figure S3.** Remission rates (4-component definitions) each using different lung function criteria, stratified by $\beta_2$-adrenergic receptor Arg16Gly genotype [arginine homozygotes (Arg/Arg), arginine/glycine heterozygotes (Arg/Gly), and glycine homozygotes (Gly/Gly)] and for the overall cohort. Data are presented as percentages of the total.

**Table S2.** Clinical remission rates and secondary failures

| | 12 months | | | | 24 months | | | | Sustained remission (12 → 24 months) | | | | Secondary failures* | | | |
|---|---|---|---|---|---|---|---|---|---|---|---|---|---|---|---|---|
| **Remission definitions** | ***Arg/Arg* (n=58)** | ***Arg/Gly* (n=29)** | ***Gly/Gly* (n=15)** | **Total (n=102)** | ***Arg/Arg* (n=58)** | ***Arg/Gly* (n=29)** | ***Gly/Gly* (n=15)** | **Total (n=102)** | ***Arg/Arg* (n=58)** | ***Arg/Gly* (n=29)** | ***Gly/Gly* (n=15)** | **Total (n=102)** | ***Arg/Arg* (n=58)** | ***Arg/Gly* (n=29)** | ***Gly/Gly* (n=15)** | ***P*-value** |
| No annual severe exacerbations + no maintenance OCS use in the past 6 months + ACT ≥20 | 23 (39.7) | 14 (48.3) | 10 (66.7) | 47 (46.1) | 28 (48.3) | 19 (65.5) | 12 (80) | 59 (57.8) | 17 (29.3) | 10 (34.5) | 8 (53.3) | 35 (34.3) | 6 (10.3) | 4 (13.8) | 2 (13.3) | 0.8767 |
| No annual severe exacerbations + no maintenance OCS use in the past 6 months + ACT ≥20 + $FEV_1$ change ≤ 5% from baseline | 20 (34.5) | 13 (44.8) | 9 (60) | 42 (41.2) | 22 (37.9) | 17 (58.6) | 11 (73.3) | 50 (49) | 14 (24.1) | 10 (34.5) | 7 (46.7) | 31 (30.4) | 6 (10.3) | 3 (10.3) | 2 (13.3) | 0.9423 |
| No annual severe exacerbations + no maintenance OCS use in the past 6 months + ACT ≥20 + $FEV_1$ ≥80% | 15 (25.9) | 11 (37.9) | 7 (46.7) | 33 (32.4) | 22 (37.9) | 13 (44.8) | 10 (66.7) | 45 (44.1) | 13 (22.4) | 9 (31) | 5 (33.3) | 27 (26.5) | 2 (3.4) | 2 (6.9) | 2 (13.3) | 0.3364 |
| No annual severe exacerbations + no maintenance OCS use in the past 6 months + ACT ≥20 + $FEV_1$ +100mL | 18 (31) | 12 (41.7) | 7 (46.7) | 37 (36.3) | 17 (29.3) | 15 (51.7) | 10 (66.7) | 42 (41.2) | 12 (20.7) | 9 (31) | 5 (33.3) | 26 (25.5) | 6 (10.3) | 3 (10.3) | 2 (13.3) | 0.9423 |
| No annual severe exacerbations + no maintenance OCS use in the past 6 months + ACT ≥20 + $FEV_1$ decline < 100mL from the best value of the first 12 months | - | - | - | - | 13 (22.4) | 7 (24.1) | 9 (60) | 29 (28.4) | 9 (15.5) | 6 (20.7) | 5 (33.3) | 20 (19.6) | - | - | - | - |

*Clinical remission was initially achieved at 12 months but was subsequently lost (due to either severe exacerbation, need for maintenance OCS, ACT <20, or loss of the lung function parameter specific to each remission definition) at any follow-up visit after month 12.
The Cochran-Armitage test for trend was used to compare the rates of secondary remission failure across genotypes.

*Abbreviations: ACT, Asthma Control Test; Arg, arginine; $FEV_1$, forced expiratory volume in one second; Gly, glycine; OCS, oral corticosteroids (prednisone equivalent dose).*

**Table S3.** Baseline predictors of clinical remission and sustained remission according to the *main definition* (3-component).

| ***Main definition:*** No annual severe exacerbations + no maintenance OCS use in the past 6 months + ACT ≥20 | | | | | | | | | |
|---|---|---|---|---|---|---|---|---|---|
| **Variable** | **12 months** | | | **24 months** | | | **Sustained remission (12 → 24 months)** | | |
| | **Adjusted OR** | **95% CI** | ***P*-value** | **Adjusted OR** | **95% CI** | ***P*-value** | **Adjusted OR** | **95% CI** | ***P*-value** |
| Centres | — | — | 0.159 | — | — | 0.054 | — | — | 0.136 |
| Centre: Bari | 2.811 | 0.391 – 20.228 | 0.305 | 1.734 | 0.212 – 14.155 | 0.607 | 2.645 | 0.339 – 20.626 | 0.353 |
| Centre: Catania | 1.037 | 0.191 – 5.628 | 0.967 | 0.280 | 0.043 – 1.814 | 0.182 | 1.000 | 0.173 – 5.792 | 1.000 |
| Centre: Foggia | 16.544 | 0.851 – 321.680 | 0.064 | 3.821 | 0.201 – 72.772 | 0.373 | 19.719 | 0.919 – 423.276 | 0.057 |
| Gly allele | 1.796 | 0.853 – 3.782 | 0.123 | 2.862 | 1.203 – 6.811 | **0.017** | 1.528 | 0.689 – 3.389 | 0.297 |
| Female | 1.115 | 0.334 – 3.725 | 0.860 | 0.611 | 0.163 – 2.286 | 0.464 | 1.770 | 0.460 – 6.804 | 0.406 |
| BMI | 0.985 | 0.891 – 1.088 | 0.762 | 1.038 | 0.938 – 1.148 | 0.475 | 1.021 | 0.916 – 1.138 | 0.707 |
| Age (years) | 0.975 | 0.927 – 1.026 | 0.333 | 0.961 | 0.910 – 1.015 | 0.156 | 0.964 | 0.911 – 1.020 | 0.203 |
| Current/ex-smoker | 0.500 | 0.165 – 1.511 | 0.219 | 0.526 | 0.171 – 1.625 | 0.264 | 0.384 | 0.120 – 1.229 | 0.107 |
| Exacerbations (n/year) | 0.897 | 0.747 – 1.078 | 0.248 | 0.912 | 0.763 – 1.091 | 0.314 | 0.829 | 0.665 – 1.033 | 0.095 |
| LAMA | 0.664 | 0.198 – 2.234 | 0.509 | 1.546 | 0.471 – 5.077 | 0.472 | 0.892 | 0.241 – 3.303 | 0.864 |
| As-needed SABA | 0.230 | 0.066 – 0.802 | **0.021** | 0.258 | 0.067 – 0.986 | **0.048** | 0.133 | 0.034 – 0.521 | **0.004** |
| OCS (mg/day) | 0.970 | 0.917 – 1.026 | 0.289 | 0.974 | 0.919 – 1.031 | 0.357 | 0.949 | 0.892 – 1.010 | 0.099 |
| LTRA | 2.097 | 0.646 – 6.804 | 0.218 | 1.239 | 0.396 – 3.872 | 0.713 | 1.307 | 0.360 – 4.747 | 0.684 |
| Blood eosinophils (cells/μL) | 1.002 | 1.000 – 1.003 | **0.022** | 1.001 | 1.000 – 1.002 | 0.089 | 1.002 | 1.000 – 1.003 | **0.013** |

*P*-values highlighted in bold are statistically significant.

*Abbreviations: BMI, body mass index; LAMA, long-acting muscarinic antagonist; LTRA, Leukotriene receptor antagonists; mAb, monoclonal antibody; Gly, glycine; OCS, oral corticosteroids (prednisone equivalent dose); SABA, short-acting $\beta_2$-agonist.*

**Table S4.** Baseline predictors of clinical remission and sustained remission according to the *secondary definition* (4-component).

| ***Secondary definition***: No annual severe exacerbations + no maintenance OCS use in the past 6 months + ACT ≥20 + $FEV_1$ decline ≤5% from baseline | | | | | | | | | |
|---|---|---|---|---|---|---|---|---|---|
| **Variable** | **12 months** | | | **24 months** | | | **Sustained remission (12 → 24 months)** | | |
| | **Adjusted OR** | **95% CI** | ***P*-value** | **Adjusted OR** | **95% CI** | ***P*-value** | **Adjusted OR** | **95% CI** | ***P*-value** |
| Centres | — | — | 0.108 | — | — | 0.094 | — | — | 0.062 |
| Centre: Bari | 1.596 | 0.233 – 10.952 | 0.634 | 0.995 | 0.135 – 7.342 | 0.996 | 1.769 | 0.237 – 13.226 | 0.578 |
| Centre: Catania | 0.591 | 0.108 – 3.232 | 0.544 | 0.289 | 0.047 – 1.790 | 0.182 | 0.516 | 0.088 – 3.025 | 0.463 |
| Centre: Foggia | 15.574 | 0.785 – 308.999 | 0.072 | 5.504 | 0.300 – 101.064 | 0.251 | 15.995 | 0.807 – 317.136 | 0.069 |
| Gly allele | 1.987 | 0.937 – 4.214 | 0.074 | 3.055 | 1.341 – 6.964 | **0.008** | 1.973 | 0.885 – 4.402 | 0.097 |
| Female | 1.290 | 0.384 – 4.337 | 0.680 | 1.272 | 0.360 – 4.492 | 0.709 | 1.606 | 0.428 – 6.024 | 0.482 |
| BMI | 0.969 | 0.877 – 1.070 | 0.534 | 1.010 | 0.915 – 1.115 | 0.846 | 0.362 | 0.113 – 1.156 | 0.086 |
| Age (years) | 0.996 | 0.947 – 1.047 | 0.878 | 1.003 | 0.955 – 1.054 | 0.892 | 0.992 | 0.941 – 1.046 | 0.762 |
| Current/ex-smoker | 0.514 | 0.171 – 1.549 | 0.237 | 0.401 | 0.133 – 1.212 | 0.105 | 0.362 | 0.113 – 1.156 | 0.086 |
| Exacerbations (n/year) | 0.928 | 0.773 – 1.115 | 0.426 | 0.981 | 0.823 – 1.170 | 0.830 | 0.952 | 0.779 – 1.162 | 0.626 |
| LAMA | 0.754 | 0.220 – 2.589 | 0.654 | 1.647 | 0.499 – 5.442 | 0.413 | 0.764 | 0.204 – 2.861 | 0.690 |
| As-needed SABA | 0.338 | 0.097 – 1.178 | 0.089 | 0.324 | 0.091 – 1.159 | 0.083 | 0.228 | 0.062 – 0.846 | **0.027** |
| OCS (mg/day) | 0.971 | 0.918 – 1.027 | 0.298 | 0.971 | 0.919 – 1.026 | 0.299 | 0.963 | 0.906 – 1.023 | 0.224 |
| LTRA | 2.242 | 0.689 – 7.298 | 0.180 | 1.128 | 0.365 – 3.485 | 0.834 | 1.953 | 0.557 – 6.850 | 0.296 |
| Blood eosinophils (cells/µL) | 1.001 | 1.000 – 1.003 | **0.036** | 1.001 | 1.000 – 1.002 | 0.239 | 1.001 | 1.000 – 1.003 | 0.051 |

*P*-values highlighted in bold are statistically significant.

*Abbreviations: BMI, body mass index; LAMA, long-acting muscarinic antagonist; LTRA, Leukotriene receptor antagonists; mAb, monoclonal antibody; Gly, glycine; OCS, oral corticosteroids (prednisone equivalent dose); SABA, short-acting $\beta_2$-agonist.*

## REFERENCES


1. Martinez FD, Graves PE, Baldini M, Solomon S, Erickson R. Association between genetic polymorphisms of the beta2-adrenoceptor and response to albuterol in children with and without a history of wheezing. *J Clin Invest*. Dec 15 1997;100(12):3184-8. doi:10.1172/jci119874
2. Wechsler ME, Kunselman SJ, Chinchilli VM, et al. Effect of beta2-adrenergic receptor polymorphism on response to longacting beta2 agonist in asthma (LARGE trial): a genotype-stratified, randomised, placebo-controlled, crossover trial. *Lancet*. Nov 21 2009;374(9703):1754-64. doi:10.1016/s0140-6736(09)61492-6
3. Contopoulos-Ioannidis DG, Manoli EN, Ioannidis JP. Meta-analysis of the association of beta2-adrenergic receptor polymorphisms with asthma phenotypes. *J Allergy Clin Immunol*. May 2005;115(5):963-72. doi:10.1016/j.jaci.2004.12.1119
